# Sorafenib-Loaded Long-Circulating Nanoliposomes for Liver Cancer Therapy

**DOI:** 10.1155/2020/1351046

**Published:** 2020-05-06

**Authors:** Haiwei Ye, Liping Zhou, Haili Jin, Yunhua Chen, Die Cheng, Ying Jiang

**Affiliations:** Chemical Pharmaceutical Research Institute, Taizhou Vocational & Technical College, Taizhou 318000, P. R., China

## Abstract

A type of sorafenib- (SOR-) loaded long-circulating nanoliposome was constructed, and the targeting performance and antitumor effects of the prepared liposome were evaluated in the present study. Polyethylene glycol- (PEG-) modified long-circulating nanoliposomes (LC-NPs) were designed and prepared using reverse evaporation, and the LC-NPs were used for delivering sorafenib (LC-PEG-SOR-NPs). Then, the anti-VEGFR antibody as a targeting moiety was chemically coupled with LC-PEG-SOR-NPs to form liver cancer-targeted nanoliposomes (anti-VEGFR-LC-PEG-SOR-NPs). The drug entrapment and loading efficiency were measured. And the cancer-targeting performance and therapeutic efficiency were evaluated both *in vitro* and *in vivo*. The anti-VEGFR-LC-PEG-SOR-NPs with an average of 119.8 ± 4.2 nm showed a uniform spherical structure. The drug entrapment and loading efficiency were 92.5% and 18.5%, respectively. The killing efficiency of anti-VEGFR-LC-PEG-SOR-NPs was up to 18% after incubating with liver cancer cells for 72 h. Furthermore, the anti-VEGFR-LC-PEG-SOR-NPs could actively target at the tumor region and could efficiently inhibit tumor growth with negligible side effects. This newly designed nanoliposomes had desirable dispersibility, high drug entrapment efficiency, tumor targeting and therapeutic efficiency, and good safety. As a biocompatible nanocomposite, it was promising to become a novel and useful tumor-targeting nanodrug for liver cancer therapy.

## 1. Introduction

As the sixth most common tumor worldwide, liver cancer ranks the third in cancer mortality, while 80% of patients were diagnosed in developing countries, and 44% were in China [1, 2]. Early diagnosis and surgical treatment are the best option to manage liver cancer. However, only 20% to 30% of liver cancer patients can be diagnosed early according to the statistical data [3, 4]. Most patients in the middle and late stages have to choose chemotherapy, radiotherapy, and interventional therapy, but those chemical drugs also damage normal liver tissues [5, 6]. At present, how to improve the targeting effect of drugs for liver cancer cells, extend the effective time of drugs, and reduce the damage to normal tissues has become a research hotspot.

Liposomes as drug delivery system are considered to be low toxicity for normal tissues [7]. However, after intravenous injection, conventional liposomes can be easily recognized by the reticuloendothelial system and quickly cleared from the circulation, resulting in limited application. The surface modification of liposomes can effectively improve their efficacy and biocompatibility [8, 9]. For example, PEGylated liposomes can avoid the recognition of the reticuloendothelial system and extend the circulation time in the body [10, 11]. The specific ligand (such as antibodies, peptides, hormones, and sugars) modification on the liposome surface can specifically target at cells [12, 13]. Based on the existing findings, targeted therapy plays an important role in patients with advanced liver cancer, such as targeted therapy with sorafenib (SOR) [14]. SOR is a multitargeted tyrosine kinase inhibitor approved by the European EMEA and US FDA for the treatment of liver cancer in 2007 [15]. Sorafenib inhibits the RAS/RAF/MEK/ERK signaling pathway by inhibiting the activity of RAF, thereby directly suppresses tumor cell proliferation, and induces apoptosis of cancer cells [16]. On the other hand, SOR can also inhibit neovascularization and cut off the supply of tumor nutrition, thereby inhibiting tumor growth and metastasis [17]. And SOR as a chemical drug has a broad-spectrum antitumor effects and is commonly used in the treatment of liver and kidney cancer. However, the side effects, such as cardiotoxicity, leukopenia, and liver damage, have seriously affected the clinical therapeutic effects of SOR in cancer treatment [18, 19]. Developing a specific delivery system of small molecule drugs to the tumor site and combining the targeted therapy can not only suppress cancer development but also avoid the potential side effects. Therefore, developing a new delivery system of tumor-targeting drug is urgently needed for clinical application of targeted therapy. The high expression of vascular endothelial growth factor receptor (VEGFR) in liver cancer plays a key role in neovascularization, tumor growth, and metastasis [20]. Therefore, VEGF and its receptor VEGFR have become the targets for the development of liver cancer drugs.

In this study, long-circulating nanoliposomes (LC-NPs) modified with distearyl phosphatidylethanolamine-polyethylene glycol (DSPE-PEG) is used for loading the liver-targeting drug SOR by reverse evaporation, forming the long-circulating liver-targeting sorafenib nanoliposomes (LC-PEG-SOR-NPs). Then the anti-VEGFR antibody as a targeting moiety was chemically coupled with LC-PEG-SOR-NPs to form the liver cancer-targeted nanoliposomes (anti-VEGFR-LC-PEG-SOR-NPs). Our results showed that anti-VEGFR-LC-PEG-SOR-NPs had good specificity and affinity for tumor cells, and the liposomes were expected to become a novel type of antitumor drug carrier.

## 2. Materials and Methods

### 2.1. Main Reagents and Instruments

Experimental SPF-level Balb/c-nu mice were purchased from Shanghai Slack Laboratory Animal Co., Ltd.; DMEM medium, fetal bovine serum, and trypsin were purchased from Gibco; VEGFR antibody was purchased from eBioscience; DSPE-PEG was obtained from Avanti, USA; carboxymethyl chitosan cetyl quaternary ammonium salt (HQCMC), Prussian blue staining kit were purchased from Solarbio; 1,2-dioleoylphosphatidylcholine (DOPC), dimethyl octadecyl epoxypropyl ammonium chloride (GHDC), cholesterol, dichloromethane, N-hydroxysuccinimide (NHS), 1-ethyl-3-(3-dimethyl ammonium propyl) ammonium bicarbonate (EDC), and other commonly used reagents were purchased from Sinopharm; and cholesterol (Chol), dichloromethane, and other commonly used reagents were purchased from Sinopharm Group. The main instruments included the BI-90Plus laser particle size analyzer/Zeta potentiometer (Brooke-Haiwen, USA), LDJ9600-1 VSM magnetic performance tester (American Digital Instrument Company), and OLYMPUS B×61 fluorescence microscope (Olympus, Japan).

### 2.2. Preparation of Anti-VEGFR-LC-PEG-SOR-NP

The anti-VEGFR-LC-PEG-SOR-NPs were prepared by reverse evaporation. Oil phase: DOPC (the matrix material), cholesterol (the skeleton material linking molecules), GHDC (the emulsifier), HQCMC (the surfactant), PEG-DSPE (the surface modification material), and a prescribed amount of SOR were co-dissolved in dichloromethane. Aqueous phase: PBS contained surfactant Tween-80, with a pH 7.4 and a concentration of 0.1 mol/L. The two phases were mixed at a volume ratio of 3 : 1. The mixed solution was ultrasonically shaken using a probe type ultrasound wave with a power of 27%, a duration of 2 s, at an interval of 1 s, for a total duration of 6 min, at 25°C, making it completely emulsified. A uniform emulsion could be formed after the ultrasound processing. Finally, the organic solvent of dichloromethane was removed by a rotary evaporator, thereby obtaining the water-soluble LC-PEG-SOR-NPs. The coupling agent 1-ethyl-3-(3-dimethylammonium propylammonium) (EDC) and N-hydroxysuccinimide (NHS) were added to the obtained LC-PEG-SOR-NP solution. Then, the VEGFR antibody was added while vortexing 30 s each time, every 30 min, for a total duration of 12 h. After that, we centrifuged to remove the free antibody and washed with water for three times. Then, we collected the supernatant and used the BCA protein kit (Solarbio) for determining the protein concentrations.

**Figure 1:**
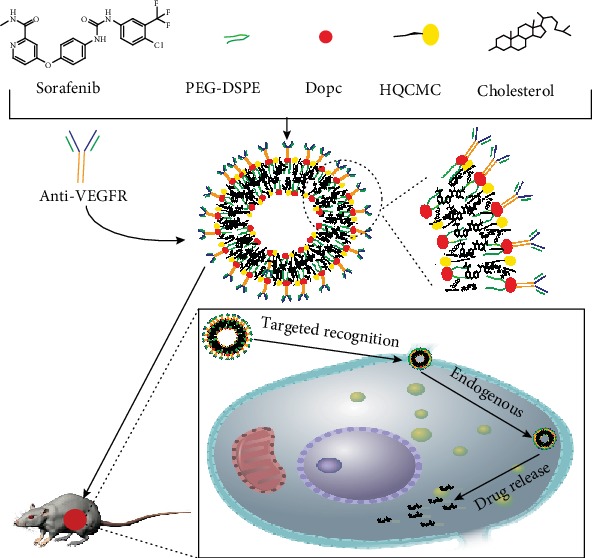
Preparation of anti-VEGFR-LC-PEG-SOR-NPs and the active targeting of drug delivery *in vivo*.

After refrigeration, the anti-VEGFR-LC-PEG-SOR-NPs were obtained. The solid content was calculated after lyophilization and diluted to a concentration of 200 *μ*g/mL with distilled water for later use. The preparation procedure is shown in Figure 1.

### 2.3. Anti-VEGFR-LC-PEG-SOR-NP Characterization and Protein Content Test

#### 2.3.1. Particle Size Analysis

10 *μ*L of anti-VEGFR-LC-PEG-SOR-NP sample was diluted to 2 mL with PBS and taken into the sample chamber of a laser particle size analyzer (Zetasizer Nano ZS90, Malvern) to measure the particle size distribution.

#### 2.3.2. Atomic Force Microscopy

The atomic-to-pitch curve could be captured by transmission atomic force microscope (AFM), and the microscopic surface of the samples could be obtained. One microliter of anti-VEGFR-LC-PEG-SOR-NP sample was diluted to 20 *μ*L with PBS. Five microliter of the diluted solution was dropped onto the mica plate and air dried for subsequent detection.

#### 2.3.3. Ultraviolet-Visible (UV-Vis) Analysis

10 *μ*L of targeted drug liposome (anti-VEGFR-LC-PEG-SOR-NP), nontargeted drug liposome (LC-PEG-SOR-NP), targeted drug-free liposome (VEGFR-LC-PEG-NP), and nontargeted drug-free liposome (LC-PEG-NP) samples were taken and diluted to 2 mL with ultrapure water (ddH_2_O), and then the absorbance spectrum was detected with the UV-Vis spectrophotometer.

#### 2.3.4. Western Blotting Analysis

The protein in anti-VEGFR-LC-PEG-SOR-NP was extracted with the RIPA solution, and the protein concentration was determined by the protein assay kit (Solarbio) and was adjusted to 3 *μ*g/*μ*L. The extracted protein solution (10 *μ*L) was mixed with 10 *μ*L of the loading buffer for sodium dodecyl sulfate-polyacrylamide gel electrophoresis (SDS-PAGE). A constant voltage of 100 V was maintained for the electrophoresis to the bottom of the separation gel. A constant current of 350 mA was transferred onto the nitrocellulose membrane after 2.5 h. After blocking with 5% skim milk powder for l h, l : 1000 diluted rabbit anti-human KDR monoclonal antibody was added, and it was shaken on a shaker overnight and rinsed with a phosphate buffer containing Tween-20 (PBST) three times for 10 min each time. Then, the 1 : 500 diluted goat anti-rabbit IgG-HRP was added, shaken on a shaker for 1 h, and rinsed with PBST three times for 10 min each time. Then, the samples were exposed with electrochemiluminescence (ECL) reagents and scanned with a scanner.

### 2.4. Determination of Anti-VEGFR-LC-PEG-SOR-NP Entrapment Efficiency and Drug Loading

One milliliter of the prepared anti-VEGFR-LC-PEG-SOR-NP was taken and centrifuged at 5000 r/min for 10 min. 0.2 mL of the centrifuged liposome sample was taken and diluted to a total volume of 0.5 mL with acetonitrile-water (60 : 40). The sample was vortexed for 30 s, added with 4 mL of tert-butyl methyl ether, and vortexed for another 1 min. After the mixture was centrifuged at 4000 r/min for 10 min, 3 mL of the supernatant was taken, and the organic solvent was removed by rotary evaporation under reduced pressure. The residue was added to 100 *μ*L of mobile phase to dissolve. Meanwhile, the uncentrifuged liposome sample was also taken and processed similarly. The concentration of the drug was determined by HPLC-UV, and the entrapment efficiency of liposomes was calculated by a plotted standard curve. The chromatographic conditions are as follows: column: VenusilMPC18 column (4.6 mm × 150 mm), 5 *μ*m; mobile phase: acetonitrile-water (60 : 40); UV-Vis detection wavelength: 227 nm; and flow rate: 1 mL/min. The detected concentrations ranged from 2 to 100 *μ*g/mL. The minimum detection limit was 30 ng.

The actual drug-loading rate was calculated as follows:

(1) $$\begin{array}{c} \text{Actual drug}-\text{loading rate }\left(\%\right)=\text{Encapsulated drug concentration}/\text{Microsphere concentration}\times 100\%. \end{array}$$

The drug entrapment efficiency was calculated as follows:

(2) $$\begin{array}{c} \text{Drug encapsulation rate }\left(\%\right)=\text{Encapsulated drug concentration}/\text{Total drug concentration}\times 100\%. \end{array}$$

### 2.5. Toxicity of Anti-VEGFR-LC-PEG-SOR-NP to Tumor Cells

In order to investigate the inhibitory ability of the anti-VEGFR-LC-PEG-SOR-NP on liver cancer tumor cells, human liver cancer Huh-7 cells in the logarithmic growth phase were selected. The cells were digested with 0.25% trypsin, inoculated into a 96-well cell culture plate at a concentration of 3000 cells/well, and cultured in a 5% CO_2_ incubator at 37°C for 12 h. The targeted drug liposome VEGFR-LC-PEG-SOR-NP, targeted drug-free liposome VEGFR-LC-PEG-NP, nontargeted drug liposome LC-PEG-SOR-NP, and SOR were dissolved in PBS (pH 7.4) and added to the 96-well plate with cells at a concentration of 200 *μ*g/mL. The cells were cultured for 1, 3, 6, 12, 24, 48, and 72 h, respectively. Cell proliferation was measured by the cell viability assay (MTT assay). Result are expressed as mean ± SD (*n* = 6).

### 2.6. Antitumor Effects of Anti-VEGFR-LC-PEG-SOR-NP *In Vivo*

#### 2.6.1. Establishment of Animal Models

The Huh-7 cells in a logarithmic growth phase were used, trypsinized and washed three times with precooled PBS, adjusted to a cell concentration of 5 × 10^6^ cells/mL, and stored at 4°C for later use. 100 *μ*L of Huh-7 cells were inoculated from the right hind limb of male BALB/c nude mice (8 weeks old). After 10 days, if a lump could be palpable at this site, the animal model would be established successfully.

#### 2.6.2. Grouping of Tumor-Bearing Nude Mice

Twenty-four nude mice bearing tumors with a uniform tumor size were randomly divided into four groups with six animals in each group. The groups were divided as a targeted drug group with anti-VEGFR-LC-PEG-SOR-NP, a nontargeted drug group with LC-PEG-SOR-NP, a SOR group with the same concentration, and a negative control group with PBS.

#### 2.6.3. The Circulation Dynamic Characteristic of Anti-VEGFR-LC-PEG-SOR-NP *In Vivo*

The anti-VEGFR-LC-PEG-SOR-NP was labeled with Cy5.5 and intravenously injected to the tumor-bearing mice (*n* = 3). Then, blood drops were taken to detect the fluorescence intensity (Ex/Em = 675/693 nm) at 0.5, 1, 2, 4, 8, and 16 hours later.

#### 2.6.4. Drug Effect Experiments

Four groups of mice were injected with VEGFR-LC-PEG-SOR-NP, LC-PEG-SOR-NP, SOR, and PBS in situ. Each animal was administered with 100 *μ*L each time, while the drug group contained 200 *μ*g of SOR. The dose was given every other day for two consecutive weeks. The conditions of the mice were carefully observed. The mice were weighed every other day, and the tumor size was measured regularly with a vernier caliper. The relative volume of the tumor was calculated as follows:

(3) $$\begin{array}{c} \text{Tumor volume}=\text{Long tumor}\times \text{Tumor width}\times \text{Tumor width}/2.\\ \text{Tumor relative volume}=\text{Tumor volume on day }N/\text{Tumor volume on day }0 \end{array}$$

### 2.7. Statistical Analysis

All statistical analysis in this study was performed using the SPSS 21.0 software. The significance of the difference between more than two groups was evaluated by the one-way analysis of variance (ANOVA) followed by the post hoc multiple comparison with Tukey's test. Pearson's correlation analysis was performed to investigate the association. *P* value < 0.05 was considered significant.

## 3. Results

### 3.1. Anti-VEGFR-LC-PEG-SOR-NP Characterization

**Figure 2:**
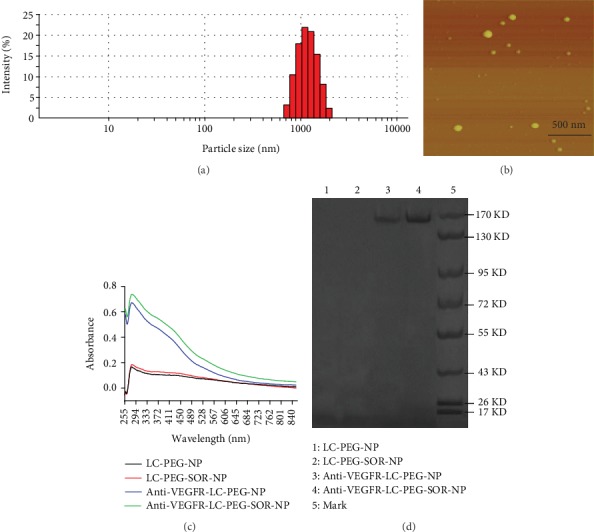
Anti-VEGFR-LC-PEG-SOR-NP characterization and protein content test. (a) Particle size test of anti-VEGFR-LC-PEG-SOR-NPs. (b) Atomic force test of anti-VEGFR-LC-PEG-SOR-NPs. (c) UV-Vis absorption spectrum of long-circulating nanoliposomes. (d) Protein electropherogram of long-circulating nanoliposomes. Representative results from three independent experiments are shown.

The average particle size of anti-VEGFR-LC-PEG-SOR-NP was 119.8 ± 4.2 nm (Figure 2(a)), which was measured by a particle size analyzer. Such small size implied that it was suitable for *in vivo* application. Moreover, the particle sizes of the prepared anti-VEGFR-LC-PEG-SOR-NPs ranged from 70.89 to 198.0 nm, with a polydispersity index (PDI) of 0.268. The centralized distribution of the particle size indicated that the particles were relatively uniform. According to the atomic force detection (Figure 2(b)), the particle sizes concentrated around 119.8 nm. The cationic polymer liposomes tended to be in a spherical shape, and the dispersion was good.

Figure 2(c) presented the UV-Vis absorption spectra of the anti-VEGFR-LC-PEG-SOR-NP, LC-PEG-SOR-NP, anti-VEGFR-LC-PEG-NP, and LC-PEG-NP. The protein exhibited a characteristic absorption peak at 280 nm in the ultraviolet spectrum. Therefore, compared to the nontargeted LC-PEG-SOR-NP and LC-PEG-NP, the targeted anti-VEGFR-LC-PEG-SOR-NP and anti-VEGFR-LC-PEG-NP showed absorption peaks at around 280 nm. It indicated that the targeting moiety of long-circulating nanoliposomes was successfully conjugated to the liposomes. The protein electropherograms of anti-VEGFR-LC-PEG-SOR-NP, LC-PEG-SOR-NP, anti-VEGFR-LC-PEG-NP, and LC-PEG-NP (Figure 2(d)) showed that the bands appeared between 130 and 170 kD, which indicated the anti-VEGFR antibody molecular. It further confirmed that the VEGFR antibody was modified on the surface of long-circulating nanoliposomes. And the binding efficiency of the antibody loading onto the liposomes was ~23.1% according to our calculation.

### 3.2. Drug-Loading Efficiency of Anti-VEGFR-LC-PEG-SOR-NP and Targeting Performance

**Figure 3:**
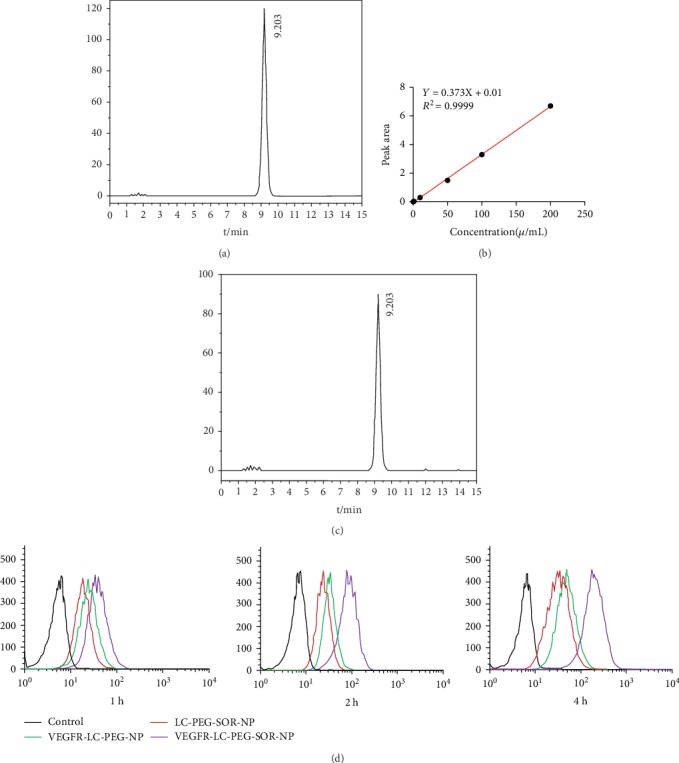
The drug-loading efficiency and targeting performance of nanoliposomes. (a) The HPLC chromatogram of standard SOR drugs. (b) The standard curve of SOR drug content. (c) The HPLC chromatogram of SOR drug content in VEGFR-LC-PEG-SOR-NP. (d) The cellular uptake of nanoliposomes determined by flow cytometry. Representative results from three independent experiments are shown.

Figure 3(a) presents the standard spectrum of SOR drugs showing the retention time at 9.2 min and a good separation effect. The standard curve is shown in Figure 3(b), with the drug concentration as the horizontal coordinates and the peak area as the vertical ordinate. The standard curve equation could be obtained as *Y* = 0.373*X* + 0.010, *R*^2^ = 0.9999, with a good linear relationship, where *Y* is the peak area of SOR drugs and *X* is the concentration of SOR drugs. The HPLC chromatogram of anti-VEGFR-LC-PEG-SOR-NP samples is shown in Figure 3(c). The sample concentration was calculated from the detected sample peak area and the standard curve. The anti-VEGFR-LC-PEG-SOR-NP sample had a SOR concentration of 37 *μ*g/mL, and the total concentration of SOR drugs was 40 *μ*g/mL. The concentration of the prepared long-circulating nanoliposomes was 200 *μ*g/mL. The entrapment efficiency of the long-circulating nanoliposomes was 92.5%, while the drug loading of the long-circulating nanoliposomes was 18.5%.

The nanoliposomes were labeled with Cy5.5 and incubated with Huh-7 cells for 1, 2, and 4 hours. According to the flow cytometer analysis (Figure 3(d)), either anti-VEGFR-LC-PEG-NP or anti-VEGFR-LC-PEG-SOR-NP showed more obviously and time-dependent accumulation within cells than other groups due to the antibody targeting property.

### 3.3. The Cytotoxic of Nanoliposomes

**Figure 4:**
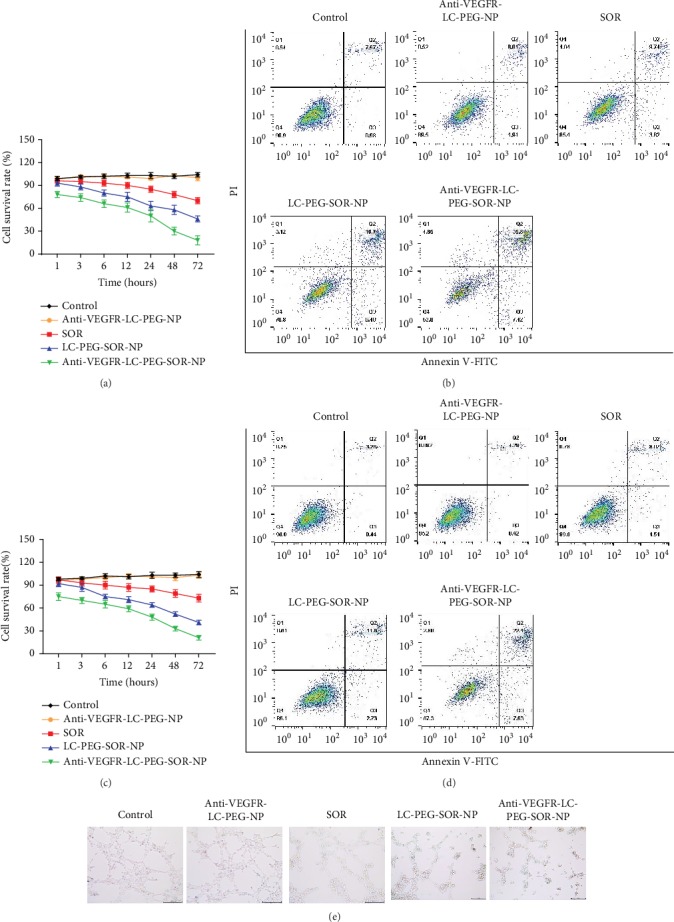
The killing effects of different sample treatment for Huh-7 and HUVEC cells. (a) Cell viability of Huh-7 cells determined by CCK8 assay following different treatments. (b) Cell apoptosis rate of Huh-7 cells determined by flow cytometry following different treatments. (c) Cell viability of HUVEC cells determined by CCK8 assay following different treatments. (d) Cell apoptosis rate of HUVEC cells determined by flow cytometry following different treatments. (e) Images of angiogenesis of HUVEC cells following different treatments. The data are presented as the mean ± SD. ^∗^*P* < 0.05, ^∗∗^*P* < 0.01 vs. the control group; ^#^*P* < 0.05, ^##^*P* < 0.01 vs. the anti-VEGFR-LC-PEG-NP group; ^&^*P* < 0.05, ^&&^*P* < 0.01 vs. the SOR group; ^$^*P* < 0.05, ^$$^*P* < 0.01 vs. the LC-PEG-SOR-NP group.

The killing efficiency of nanodrugs for tumor cells was detected by the MTT assay. As shown in Figure 4(a), the anti-VEGFR-LC-PEG-SOR-NP significantly inhibited the survival of liver cancer cells. As the culture time extended, the survival rate of cancer cells gradually decreased, and the survival rates were only approximately 30% and 18% after 48 h and 72 h incubation. On the contrary, the drug-free nanoliposome (anti-VEGFR-LC-PEG-NP) hardly affected the cell activity, and the survival rate was approximately 98%. The same concentration of SOR had no obvious killing effect on Huh-7 cells, and the survival rate was maintained above 70%, while the nontargeted liposome LC-PEG-SOR-NP containing the same concentration of SOR showed more cytotoxic to Huh-7 cells than that of the SOR treatment group. However, the killing effect was also not as high as that of anti-VEGFR-LC-PEG-SOR-NP, and the survival rate was over 46% after 72 h. The flow cytometry analysis further proved the anti-VEGFR-LC-PEG-SOR-NP to trigger more percentage (43.22%) of apoptosis in cancer cells which was the Annexin V-FITC positive staining (Figure 4(b)). Since tumor angiogenesis was vital for tumor cells proliferation and metastasis, and it was well known that SOR suppressed tumor growth by inhibiting angiogenesis and destroying tumor microvessel [21], thus, we further detected the cytotoxic effect of nanoliposomes for human umbilical vein endothelial cells (HUVEC). The MTT results showed a similar trend to that of Huh-7 cells (Figure 4(c)). The flow cytometry analysis further proved the anti-VEGFR-LC-PEG-SOR-NP triggered more cellular apoptosis (29.93%) in endothelial cells (Figure 4(d)). More importantly, the anti-VEGFR-LC-PEG-SOR-NP remarkably inhibited the HUVEC tube formation on a basement membrane substrate (Figure 4(e)), which confirmed that SOR-loaded targeting nanoliposomes not only suppressed the cancer cell proliferation but also inhibited angiogenesis. These results indicated that even at the concentration of 200 *μ*g/mL, the drug-free liposome did not affect the cell viability and showed good biocompatibility to be a drug carrier. On the other hand, although SOR had weak lethality for Huh-7 cells, the killing efficiency remarkably enhanced after SOR loaded into the targeted nanoliposomes.

### 3.4. Tumor Suppression Effect of Nanoliposomes

**Figure 5:**
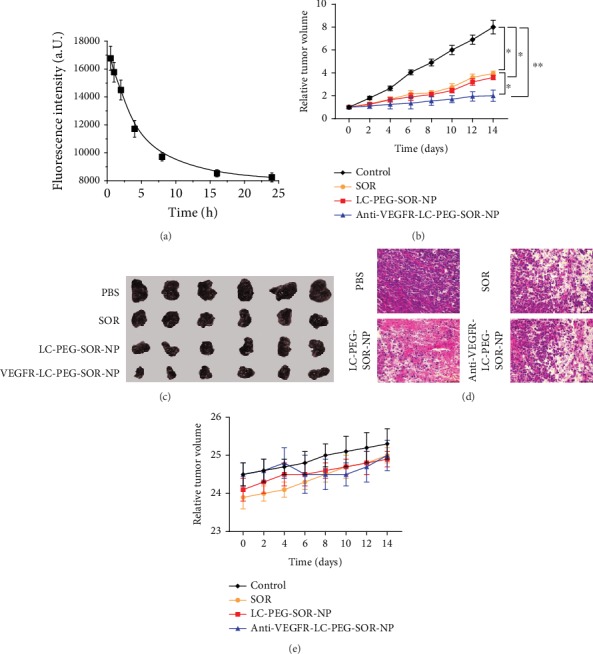
The tumor suppression effect *in vivo*. (a) Monitoring of the fluorescence-labeled nanoliposomes after intravenous injection. (b) The relative volume curve of mice in each treatment group. (c) The photographs of collected tumor tissues. (d) H&E staining of the tumor slices. (e) The weight change curves of mice in each treatment group. The data are presented as the mean ± SD. ^∗^*P* < 0.05, ^∗∗^*P* < 0.01.

**Figure 6:**
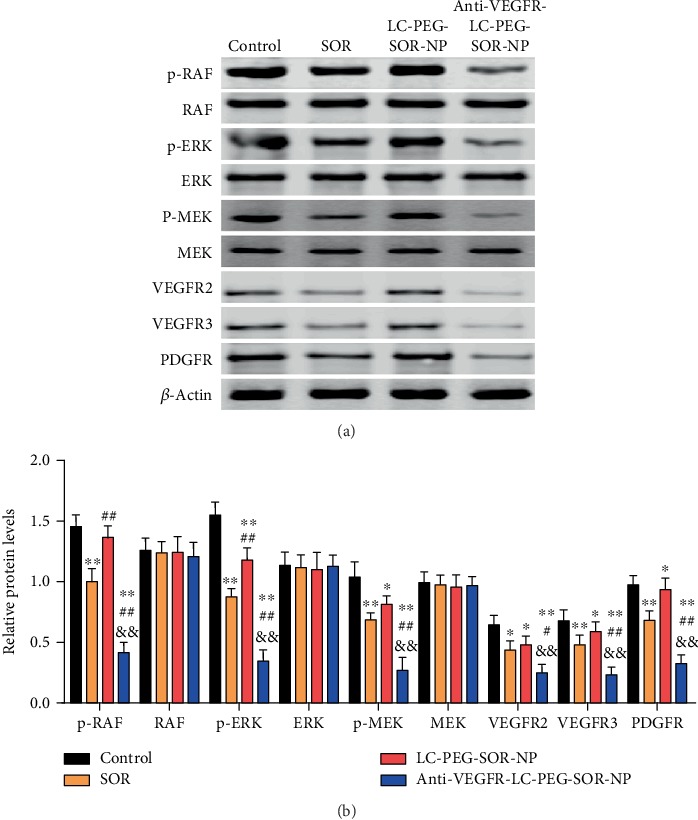
The expression of SOR target-related proteins, including p-RAF, RAF, p-ERK, ERK, p-MEK, MEK, VEGFR2, VEGFR3, and PDGFR. Results are expressed as mean ± SD (*n* = 3).^∗^*P* < 0.05, ^∗∗^*P* < 0.01 vs. the control group; ^#^*P* < 0.05, ^##^*P* < 0.01 vs. the SOR group; ^&^*P* < 0.05, ^&&^*P* < 0.01 vs. the LC-PEG-SOR-NP group.

Firstly, we evaluated the circulation time of the fluorescence-labeled anti-VEGFR-LC-PEG-SOR-NP after being intravenously injected into the mice. The half time of nanoliposomes was nearly 10 hours, which revealed their long circulation capability (Figure 5(a)). The tumor growth curve of mice model showed that compared with the PBS treatment group, the groups with the same concentration of SOR and LC-PEG-SOR-NPs could significantly suppress the tumor growth (*P* < 0.05). However, the inhibitory effect of the LC-PEG-SOR-NP group was more significant than that of the SOR group (Figure 5(b)). The underlying reason was that the LC-PEG-SOR-NPs could stay longer in the blood circulation of mice, leading to more obvious antitumor effect. The anti-VEGFR-LC-PEG-SOR-NP group could more significantly suppress tumor growth than the other groups (*P* < 0.05). All tumor-bearing mice were killed on the 14th day, and the photographs of respective tumor tissues showed the similar trend with growth curve (Figure 5(c)). The tumor histological slices stained by hematoxylin and eosin (H&E) showed more severe damage in anti-VEGFR-LC-PEG-SOR-NP group than that in other groups (Figure 5(d)). It was attributed to “active targeting” after being conjugated with anti-VEGFR, which enabled the nanodrugs to accurately identify the tumor cells. This indicated that the targeted long-circulating nanoliposomes could be used as a drug carrier with active targeting and sustained release. In addition, in order to examine whether the corresponding treatment caused the toxic side effects, the nude mice were weighed every other day. As shown in Figure 5(e), the bodyweight of mice in each group did not decrease and remained stable at 24.5 ± 0.6 g. This indicated that the treatment of PBS, SOR, LC-PEG-SOR-NP, and anti-VEGFR-LC-PEG-SOR-NP did not have obvious toxicity to mice. Subsequently, we investigated the expression of SOR target-related proteins in tumor tissues through western blotting. As shown in Figure 6(a), the levels of SOR target-related proteins including p-RAF, p-ERK, p-MEK, VEGFR2, VEGFR3, and PDGFR were significantly reduced. Importantly, the anti-VEGFR-LC-PEG-SOR-NP group showed more significantly lower levels of p-RAF, p-ERK, p-MEK, VEGFR2, VEGFR3, and PDGFR (Figure 6(b)). However, there was no obviously dysregulation of SOR target-related proteins in tumor tissues in response to LC-PEG-SOR-NP. These results collectively indicated that anti-VEGFR-LC-PEG-SOR-NP exhibited strong cytotoxicity on tumor cells.

## 4. Discussion

Nowadays, chemotherapy has become one of the major options for cancer therapy. However, chemical drugs often damage normal organs due to a lack of selectivity, causing serious systemic adverse reactions.

Although the nanoliposome was approved by FDA for several years, it was proved nonideal targeting characteristic and poor stable and prone to leakage *in vivo*. Positive-targeted nanoliposomes are proven to be desirable carriers for chemotherapy drug due to the following features: (1) liposomes are biocompatible and less toxic [22]; (2) nanoliposomes can easily penetrate through blood barrier and reach the target organs [23]. In addition, it can be modified with a large number of targeting molecules due to its large surface area and abundant modification sites [24]; (3) after PEG-modified long-circulating liposomes are engulfed by the liver and spleen, silicon is reduced, and they show a sufficient circulating half-life in the blood [25]; (4) after the targeting ligand is linked to liposomes, it does not affect the structural and biological properties of the drugs loaded in the liposomes [26]; and (5) benefiting from the ligand-receptor effect, it can specifically target to the tumor site, so that the drugs concentrate in the target organ and reduce distribution in other organs [27, 28].

VEGFR was a universal target overexpressed on vasculature of multiple solid tumor types and other disease models, which were specific receptors for VEGF. It was closely related to the pathogenesis and metastasis of many common tumors in the body because it would bind with VEGF and promote the neovascularization, proliferation, and migration of vascular endothelial cells. Therefore, it was not a unique targeting receptor for liver cancer cells, which was not an ideal target for the liver cancer therapy only depending on the anti-VEGFR antibody. Fortunately, the combination of nanocarriers (nanoliposomes), targeting antibodies (anti-VEGFR antibodies), and molecular targeting drugs (SOR) cleverly improved the targeting and therapeutic efficiency in this work.

Mounting evidence indicates that delivery of nanomedicine to solid tumors depends on the enhanced permeability and retention effect. The size of nanoparticle plays a pivotal role in optimizing drug delivery and therapeutic outcome [29, 30]. Our results indicated that anti-VEGFR-LC-PEG-SOR-NPs could positively target the tumor and efficiently inhibit tumor growth. Moreover, SOR as a multitarget and multikinase inhibitor can specifically inhibit serine/threonine kinase and tyrosine kinase receptor on tumor cells and tumor blood vessels [31, 32]. Most importantly, the activation of RAS-RAF-ERK-MEK-MAP kinase pathway is related to the pathogenesis of malignancies, which promotes cell proliferation and differentiation, and inhibits cell apoptosis [33, 34]. Liver cancer is a typical vascular rich tumor and VEGFR is reported to promote the development and metastasis of liver cancer through promoting lymph angiogenesis and angiogenesis [35, 36]. PDGFR is found to regulate cell proliferation, differentiation, growth, and development [37]. The inhibition of nanodrugs on these proteins explains the cytotoxicity of tumor cells treated with anti-VEGFR-LC-PEG-SOR-NPs. In summary, we designed the targeting nanoliposomes (anti-VEGFR-LC-PEG-SOR-NPs) that hardly show side effects on mice and are promising for clinical application transformation.

## 5. Conclusion

In summary, the prepared liver cancer-targeted anti-VEGFR-LC-PEG-SOR-NPs could effectively improve the *in vivo* distribution of SOR drugs and enhance its safety and efficacy *in vivo*. The prepared liposomes were expected to become a novel and effective antitumor drug carrier.

## Data Availability

All data generated or analyzed during this study are included in this published article. The data used to support the findings of this study are available from the corresponding author upon request.
